# Zein/Gum Arabic Nanoparticles for Cinnamaldehyde Delivery: Enhanced Antifungal Performance and Bread Shelf Life Extension

**DOI:** 10.1111/1750-3841.71488

**Published:** 2026-09-16

**Authors:** Mônia Sartor, Vanessa Teixeira da Rosa, Laura Smaniotto Nascimento, Alisson Steffli Thill, Fabiano Bernardi, Flávio Fonseca Veras, Juliane Elisa Welke

**Affiliations:** ^1^ Institute of Food Science and Technology (ICTA) Federal University of Rio Grande do Sul (UFRGS) Porto Alegre Rio Grande do Sul Brazil; ^2^ Graduate Program in Physics Institute of Physics, UFRGS Porto Alegre Brazil

**Keywords:** bakery products, cinnamon, encapsulation, essential oil, fungal spoilage, nanocarrier, nanostructure

## Abstract

Bread is highly susceptible to fungal spoilage, which limits its shelf life. The increasing interest in preservation strategies based on naturally derived antifungals is constrained by the instability of compounds such as cinnamaldehyde, the main component of cinnamon essential oil. This study evaluated cinnamaldehyde‐loaded zein/gum arabic nanoparticles for bread preservation, focusing on physicochemical characterization, antifungal activity, in situ performance, and storage stability. The particles, prepared by antisolvent precipitation, showed a mean particle diameter in the nanometric range (∼255 nm), a narrow size distribution (polydispersity index < 0.2), good colloidal stability (zeta potential −21.8 mV), and suitable encapsulation efficiency (71%). Scanning electron microscopy (SEM) analyses revealed spherical particles with smooth and compact surfaces. Fourier transform infrared spectroscopy (FTIR) and thermogravimetric analysis (TGA) confirmed the incorporation of cinnamaldehyde and the enhanced stability of the compound within the polymeric matrix. Small‐angle x‐ray scattering (SAXS) indicated nanoscale domains consistent with cinnamaldehyde incorporation, whereas x‐ray photoelectron spectroscopy (XPS) confirmed surface zein, supporting encapsulation. Encapsulation maintained or enhanced antifungal activity against *Penicillium citrinum*, *Penicillium roqueforti*, *Aspergillus niger*, and *Aspergillus flavus*, particularly at subinhibitory concentrations. In situ assays showed a marked delay in fungal growth and reduced contamination in bread treated with encapsulated cinnamaldehyde compared with the cinnamaldehyde control dispersion and untreated bread. Storage studies demonstrated that refrigeration preserved nanoparticle stability and antifungal activity for up to 180 days. Overall, zein/gum arabic nanoparticles provided a stable delivery system for cinnamaldehyde, enabling sustained antifungal performance in bread and offering a promising strategy for controlling fungal spoilage and extending shelf life.

## 1 Introduction

Fungal contamination is the primary factor limiting the shelf life of bakery products, leading to significant economic losses for both industry and consumers. The baking process effectively eliminates the fungal microbiota associated with raw materials. However, post‐baking recontamination is unavoidable in industrial settings, as airborne fungal spores can deposit onto products during cooling, handling, and slicing (Garcia et al. 2019). Species of *Aspergillus* and *Penicillium* are among the fungi most frequently associated with bread spoilage, causing visible mold growth and sensory deterioration. Under favorable conditions, some species may also produce mycotoxins, thereby posing a potential risk to human health (Garcia et al. 2019; Ollinger et al. 2024).

Hygienic and sanitary practices, while essential, are insufficient to ensure microbiological stability. Thus, the use of preservatives becomes critical to maintain product quality and commercial viability. The bakery industry commonly relies on synthetic preservatives, such as propionates and sorbates, to inhibit fungal growth and extend shelf life. However, these additives are increasingly viewed negatively by consumers, driving demand for clean‐label products (Pratama et al. 2026). Although *clean label* has no universally accepted regulatory definition, the term generally refers to products formulated with recognizable, minimally processed, and predominantly naturally derived ingredients, with reduced reliance on synthetic additives (Urugo et al. 2026). In addition, microbial adaptation to conventional preservatives has reduced their effectiveness (Moro et al. 2022), prompting the search for new, natural, and effective preservation strategies.

Essential oils and their major constituents have emerged as promising alternatives to synthetic preservatives. Cinnamaldehyde (CIN), the main component of cinnamon essential oil (*Cinnamomum* spp.), is recognized as safe (GRAS) by the US Food and Drug Administration for use in foods as a flavoring agent (FDA 2025, 2026a) and is likewise authorized for this purpose in the European Union (WHO 2000; Council of Europe 2012). However, this regulatory status applies to its use as a flavoring substance; CIN has not been specifically authorized as an antifungal preservative in bread. In Brazil, CIN is registered for use as an agricultural fungicide (Brasil 2021, Agência Nacional de Vigilância Sanitária—ANVISA 2022) and as a flavoring additive in animal feed (MAPA 2026). This phenylpropanoid is widely used as a flavoring agent in a range of industrial products, including beverages, baked goods, confectionery, and desserts. However, its application as an antimicrobial or antioxidant is limited, as its intense aroma and flavor may impart undesirable sensory attributes to foods (Karimirad et al. 2025).

Encapsulation technologies can overcome these limitations and enable the use of cinnamaldehyde as an antimicrobial in foods (Liu et al. 2023). The combination of zein and gum arabic has emerged as a promising strategy for encapsulating bioactive compounds in nano/microstructured systems (Gali et al. 2022; Liu et al. 2022; Song et al. 2025). Zein, an amphiphilic corn protein, forms core–shell particles through self‐assembly and hydrophobic interactions, enhancing protection and dispersibility in food matrices (Gao et al. 2025). However, its tendency to aggregate can compromise colloidal stability (Mendoza et al. 2025). In this context, gum arabic, a polysaccharide derived from *Acacia senegal* and *Acacia seyal*, acts as a stabilizing co‐polymer, improving physical stability and preventing particle aggregation (Gali et al. 2022). Both zein and gum arabic are recognized as safe (GRAS) for use in foods by the FDA (2026b, 2026c), and gum arabic is also approved as a food additive in Europe (European Food Safety Authority [EFSA] et al. 2017) and Brazil (ANVISA 2023).

The stability of nanostructured systems during storage is critical for food applications, as physicochemical changes can compromise the biological activity of active compounds. Processes, such as aggregation, flocculation, and coalescence, directly affect particle size and distribution, influencing dispersibility and interactions with the food matrix. From a technological standpoint, maintaining stability over time is essential to ensure reproducibility, consistent performance, and industrial feasibility. Stable systems also offer greater potential for scale‐up and commercialization (Rezagholizade‐Shirvan et al. 2024).

The incorporation of cinnamaldehyde into zein/gum arabic nanostructured complexes has been proposed for producing edible films to extend the shelf life of strawberries (Wang, Xue, et al. 2024), citrus fruits, and cherries (Tang et al. 2025). In bakery products, cinnamaldehyde has been used as an antifungal agent in bioplastics for bread packaging (Srisa and Harnkarnsujarit 2020; Wang et al. 2023). Additionally, its application within conventional plastic packaging, using simple systems such as impregnated filter paper, has been explored to control fungal growth during bread storage and distribution (Sun et al. 2020; Tan et al. 2025). However, the direct incorporation of cinnamaldehyde‐loaded zein/gum arabic nanoparticles into bread formulations has not been previously reported.

This study evaluates cinnamaldehyde‐loaded zein/gum arabic nanoparticles incorporated into bread formulation as an antifungal approach based predominantly on naturally derived active compounds and carrier materials. The aim was to develop, characterize, and assess the stability of these nanoparticles, as well as to evaluate their in vitro antifungal activity and effectiveness in controlling spoilage fungi in bread.

## 2 Materials and Methods

### 2.1 Reagents

Zein (Z3625, CAS 9010‐66‐6), trans‐cinnamaldehyde (CAS 14371‐10‐9, W228605, 98% purity, food grade), and gum arabic (G9752, CAS 9000‐01‐5) were purchased from Sigma‐Aldrich (St. Louis, MO, USA). Polysorbate 80 (CAS 9005‐65‐6) and polysorbate 20 (CAS 9005‐64‐5) were obtained from Synth (São Paulo, Brazil) and Êxodo Científica (São Paulo, Brazil), respectively. Ethanol (CAS 64‐17‐5) was supplied by Dinâmica (São Paulo, Brazil), and acetonitrile by J.T. Baker (New Jersey, USA).

### 2.2 Fungi

The following fungal isolates were used: *Aspergillus flavus* M19, *Aspergillus niger* UD22, *Penicillium citrinum* ITAL197, and *Penicillium roqueforti* LTQA02, obtained from the culture collection of the Food Toxicology Laboratory, Institute of Food Science and Technology (ICTA, UFRGS, Porto Alegre, Brazil). The strains were selected on the basis of their relevance as bread spoilage agents (Garcia et al. 2019). For inoculum preparation, the isolates were grown on potato dextrose agar (PDA) (Merck, Darmstadt, Germany) at 25°C for 7 days.

### 2.3 Preparation of Cinnamaldehyde Control Dispersion

Preliminary tests were conducted with polysorbate 20 and polysorbate 80 to assess their suitability for preparing the cinnamaldehyde control dispersion. Polysorbate 20 was selected because it enabled the formation of a homogeneous dispersion. Cinnamaldehyde was dispersed in a 2% (v/v) polysorbate 20 solution prepared in distilled water. The mixture was stirred at room temperature for 2 h without heating, yielding a final CIN concentration of 20 mg mL^−1^. This surfactant‐mediated dispersion was used as a control, as it did not contain the zein/gum arabic nanocarrier system and was referred to as the cinnamaldehyde control dispersion.

### 2.4 Preliminary Evaluation of the Antifungal Activity of Cinnamaldehyde

The antifungal activity of cinnamaldehyde against spoilage fungi in bakery products was preliminarily evaluated using agar diffusion and vapor‐phase assays to assess its antifungal effects with and without direct contact with fungal cells, according to Ju et al. (2018).

- For the agar diffusion test, fungal spores were added to sterilized PDA cooled to 40–45°C to obtain a final concentration of 10^3^ spores mL^−1^. Aliquots (15 mL) were poured into 90 mm Petri dishes, and after solidification, 10 µL of cinnamaldehyde were applied onto the agar surface at three equidistant points.
- For the vapor‐phase assay, 10 µL of spore suspension (adjusted to 10^3^ spores mL^−1^) were inoculated at the center of PDA plates. A sterile filter paper disc (6 mm diameter) was attached to the inner side of the lid, onto which 10 µL of cinnamaldehyde were applied, followed by sealing with parafilm.

In both assays, a 2% (v/v) polysorbate 20 solution was used as the negative control, and plates were incubated at 25°C for 7 days. Antifungal activity was evaluated by measuring inhibition zone diameters (agar diffusion) and colony diameters (vapor‐phase assay) using a digital caliper (Maxwell 150 mm, Zhejiang, China).

### 2.5 Preparation of Zein/Gum Arabic Particles Loaded With Cinnamaldehyde (ZEI/GA–CIN PT)

**FIGURE 1:**
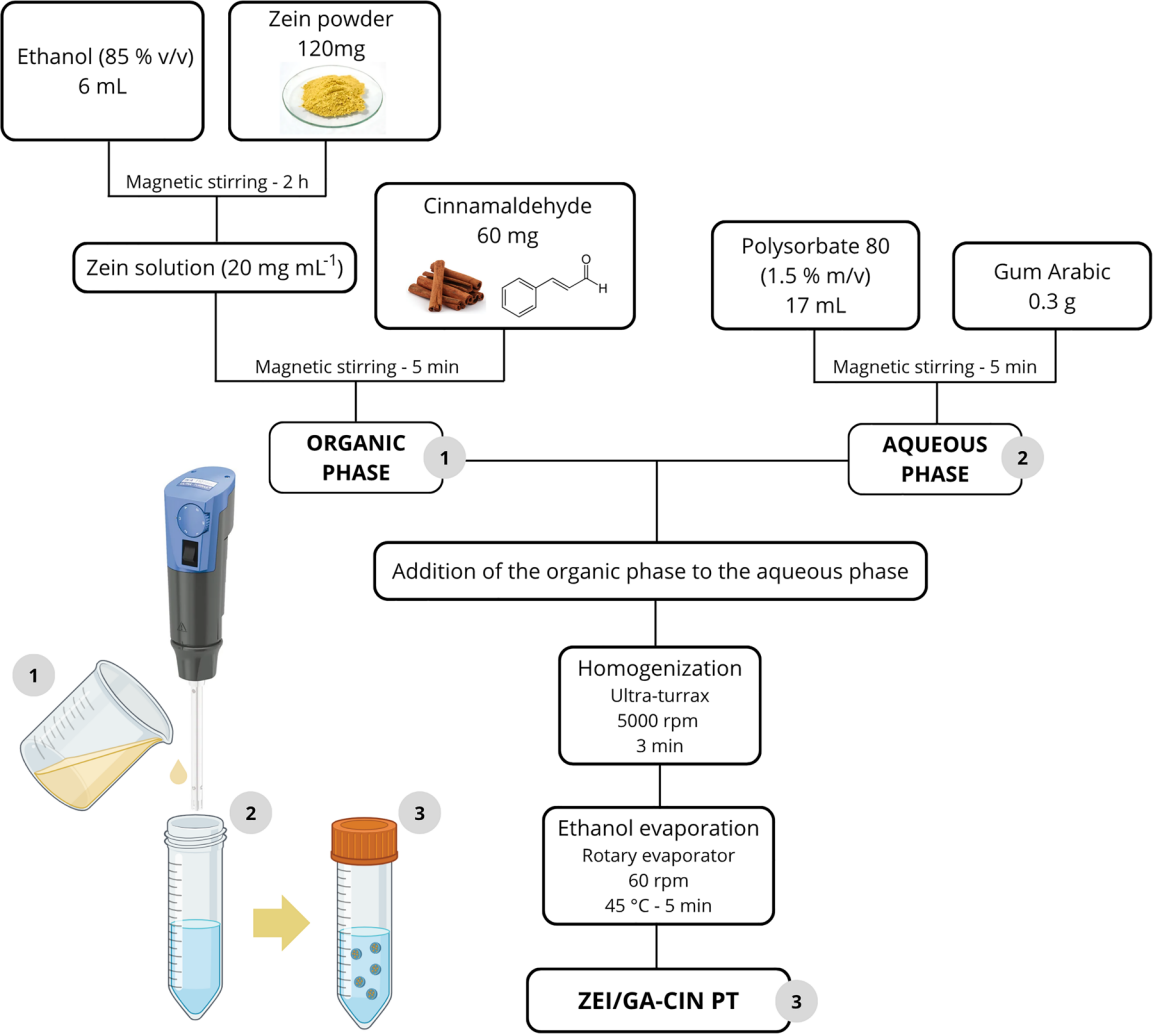
Procedure for the preparation of zein/gum arabic particles loaded with cinnamaldehyde (ZEI/GA–CIN PT) via the antisolvent precipitation method.

After confirming the antifungal potential of cinnamaldehyde, zein/gum arabic particles loaded with cinnamaldehyde (ZEI/GA–CIN PT) were prepared by antisolvent precipitation, as described by Gali et al. (2022), with modifications (Figure 1). Preliminary formulation tests were conducted to define the zein concentration and surfactant used under the conditions of the present study. The zein concentration reported by Gali et al. (2022) (25 mg mL^−1^) resulted in visible precipitation shortly after particle preparation; therefore, it was reduced to 20 mg mL^−1^. In parallel, evaluation of polysorbate 20 and polysorbate 80 showed that polysorbate 80 enabled the production of stable zein/gum arabic nanoparticle dispersions and was, therefore, selected for particle preparation.

For the organic phase, zein (120 mg) was dissolved in 6 mL of 85% (v/v) ethanol under magnetic stirring until complete dissolution (20 mg mL^−1^). The aqueous phase consisted of 17 mL of a 1.5% (w/v) polysorbate 80 solution containing 0.3 g of gum arabic.

Cinnamaldehyde (60 mg) was dissolved in the organic phase, which was then added dropwise to the aqueous phase using an Ultra‐Turrax homogenizer (5000 rpm, 3 min) to induce particle formation. The resulting dispersion was subjected to rotary evaporation (60 rpm, 45°C, 5 min) to remove ethanol, yielding a final dispersion volume of 19 mL and a cinnamaldehyde concentration of 3.16 mg mL^−1^. Blank zein/gum arabic particles (ZEI/GA PT) were prepared under the same conditions, without cinnamaldehyde (hereafter referred to as empty or unloaded nanoparticles).

### 2.6 Characterization of Zein/Gum Arabic Particles

#### 2.6.1 Mean Particle Diameter, Polydispersity Index (PDI), and Zeta Potential

ZEI/GA PT and ZEI/GA–CIN PT dispersions were diluted in ultrapure water (1:10, v/v). The mean particle diameter and PDI were determined by dynamic light scattering (DLS), and the zeta potential (*ζ*) was measured by electrophoretic light scattering, using a Litesizer DLS 700 (Anton Paar GmbH, Graz, Austria). Measurements were performed at 25°C.

#### 2.6.2 Encapsulation Efficiency and Loading Capacity

Encapsulation efficiency and loading capacity were determined according to Tópor et al. (2024). Briefly, 400 µL aliquots of ZEI/GA–CIN PT were centrifuged (10,000 rpm, 5 min) using ultrafiltration tubes (Microcon‐10 kDa, Merck, Darmstadt, Germany). The filtrate (cinnamaldehyde fraction not encapsulated within the zein/gum arabic particles) and retentate (encapsulated cinnamaldehyde) were diluted (1:10, v/v) in acetonitrile:water (80:20, v/v) and filtered through 0.22 µm PTFE syringe filters (25 mm, Allcrom, SP, Brazil).

Quantification was performed by HPLC‐DAD (Waters e2695, Milford, USA) using a C18 column (LiChrospher 100 RP‐18, 5 µm, 250 × 4 mm, Merck, Germany). The mobile phase consisted of acetonitrile:water (50:50, v/v) at a flow rate of 1 mL min^−1^, with detection at 274 nm and an injection volume of 10 µL. A calibration curve (0.8–100 µL mL^−1^) showed good linearity (*Y* = 4 × 10^8^ *x* + 95,261; *R* ^2^ = 0.9979).

Loading Capacity and Encapsulation Efficiency was calculated using the following equations:

(1) $$\begin{array}{ccc} & & \mathrm{Loading}\;\mathrm{capacity}\left(\frac{\mu g}{\mathrm{mg}}\right)\\ & & \;=\frac{\mathrm{Amount}\;\mathrm{of}\;\mathrm{encapsulated}\;\mathrm{cinnamaldehyde}}{\mathrm{Amount}\;\mathrm{of}\;\mathrm{encapsulating}\;\mathrm{agent}} \end{array}$$

(2) $$\mathrm{Encapsulation}\;\mathrm{efficiency}\left(\%\right)=\frac{Cp-Ce}{Cp}\times 100$$

where *C_p_* is the cinnamaldehyde concentration used for particle preparation, and *C_e_* is the experimentally determined cinnamaldehyde concentration in the filtrate.

#### 2.6.3 Morphological Characterization

Scanning electron microscopy (SEM) was used to evaluate the morphology of the particles using a Zeiss EVO 10 microscope (Zeiss, Oberkochen, Germany). ZEI/GA PT and ZEI/GA–CIN PT dispersions were mounted on aluminum stubs with carbon tape and dried at room temperature overnight until complete solvent evaporation. The samples were then sputter‐coated with a thin layer of gold. Images were acquired at a magnification of 50,000×, with an accelerating voltage of 10–15 kV.

#### 2.6.4 Structural and Thermal Characterization

Fourier transform infrared spectroscopy (FTIR) was used to investigate structural interactions, whereas thermogravimetric analysis (TGA) was used to assess thermal stability. Prior to analysis, samples were freeze‐dried using a freeze dryer (Liotop L101, Vitória, Brazil) under controlled conditions (−58°C and 6.7 Pa) for approximately 96 h, until complete moisture removal.

FTIR spectra were recorded using a Fourier transform infrared spectrometer (Spectrum Two, PerkinElmer, USA) equipped with a micro‐attenuated total reflectance (µATR) accessory, following ASTM E1252‐98. Spectra were acquired in the range of 4000–450 cm^−1^, with 8 scans and a resolution of 4 cm^−1^.

TGA was carried out using a thermogravimetric analyzer (Shimadzu TGA‐50, Tokyo, Japan). Approximately 10 mg of freeze‐dried sample was placed in aluminum pans and heated from 25°C to 600°C at a rate of 20°C min^−1^ under a nitrogen flow of 40 mL min^−1^, following the methodology described by da Rosa et al. (2015).

#### 2.6.5 Nanostructural Characterization

Small‐angle x‐ray scattering (SAXS) was used to investigate the nanostructural organization of ZEI/GA–CIN PT. The analysis aimed to evaluate structural organization at the nanoscale and potential changes in scattering patterns associated with the incorporation of cinnamaldehyde into the polymeric matrix. Differences in scattering profiles between cinnamaldehyde and ZEI/GA–CIN PT were used to infer changes in molecular arrangement and the formation of organized nanoscale assemblies after encapsulation.

The SAXS measurements were conducted using the Nano‐inXider (Xenocs) equipment at CNANO/UFRGS. The powder was placed in a solid sample holder and sealed with a Kapton tape. SAXS patterns were acquired with a Cu *K*α x‐ray source, a semitransparent beam stop, and a DECTRIS PILATUS3 detector. Measurements were performed in the transmission mode in a 2*θ* range of 0.00–5.25° and an exposure time of 60 s for each scan. The final SAXS pattern resulted from an average of 120 scans per sample. The FoxTrot software was used to convert symmetric two‐dimensional SAXS data into one‐dimensional data and to obtain the average between multiple scans (David and Pérez 2009).

X‐ray photoelectron spectroscopy (XPS) measurements were performed at LAMAS‐UFRGS to determine the elemental composition of the sample's surface. The sample, in the liquid form, was deposited over a Si wafer that was fixed to the sample holder and inserted into the UHV chamber. The measurements were performed with an Omicron Sphera analyzer using an Al *K*α x‐ray source (1486.6 eV) at room temperature. Long scan, C 1s, O 1s, and N 1s regions were acquired. The analyzer operated with pass energies of 50 and 10 eV and energy steps of 1.0 and 0.1 eV for the survey and high resolution regions, respectively. The dwell time in all cases was 100 ms. The base pressure during the measurements was 10^−9^ mbar.

### 2.7 Effect of Encapsulation on the In Vitro Antifungal Activity of Cinnamaldehyde

#### 2.7.1 Minimum Inhibitory Concentration (MIC) and Minimum Fungicidal Concentration (MFC)

The MIC was determined by broth microdilution according to Clinical and Laboratory Standards Institute guidelines (CLSI 2017). Spore suspensions were prepared in potato dextrose broth (PDB) and dispensed into 96‐well microplates containing serial dilutions of either ZEI/GA–CIN PT or the cinnamaldehyde control dispersion. Each well received 75 µL of spore suspension and 75 µL of the respective cinnamaldehyde preparation, resulting in a final spore concentration of 10^3^ spores mL^−1^.

Ten final concentration levels were tested for each treatment. For the cinnamaldehyde control dispersion, the final CIN concentrations were 10,000, 5000, 2500, 1250, 625, 330, 165, 82.5, 41.3, and 20.6 µg mL^−1^. For ZEI/GA–CIN PT, the final CIN concentrations were 1350, 625, 330, 165, 82.5, 41.3, 20.6, 10.3, 5.2, and 2.5 µg mL^−1^. Plates were incubated at 25°C for 7 days. The MIC was defined as the lowest concentration showing no visible fungal growth.

Wells containing only spore suspension in PDB were used as positive controls, whereas wells containing PDB with 2% (v/v) polysorbate 20 served as negative controls to confirm sterility.

The MFC was determined by transferring 10 µL from wells with no visible growth onto PDA plates. After incubation at 25°C for 7 days, the MFC was defined as the lowest concentration resulting in no colony‐forming units (CFUs).

#### 2.7.2 Effect of Encapsulation on Dose–Response Behavior and Determination of the Lethal Concentration of Cinnamaldehyde

The effect of encapsulation on the dose–response behavior of cinnamaldehyde was evaluated on the basis of mycelial growth inhibition under solid‐medium conditions. Concentrations were selected relative to previously determined MFC values (Section 2.7.1), including an initial level corresponding to 50% of the lowest MFC, covering a range from subinhibitory to fully inhibitory concentrations, and allowing the determination of lethal concentrations for each strain.

The cinnamaldehyde control dispersion and ZEI/GA–CIN PT were incorporated into molten PDA (40–45°C) to obtain the target concentrations. Aliquots (15 mL) were poured into sterile 90 mm Petri dishes, whereas plates containing only PDA served as controls. After solidification, 5 µL of spore suspension (10^3^ spores mL^−1^) was inoculated at the center of each plate.

Plates were incubated at 25°C for 7 days. Colony diameter was measured on Day 7 along three perpendicular axes using a digital caliper. All assays were performed in triplicate. Fungal growth inhibition (%) was calculated according to the following equation:

(3) $$\mathrm{Inhibition}\left(\%\right)=\frac{Dc-Dt}{Dc}\times 100$$

where *Dc* is the mean colony diameter of the control and *Dt* is the mean colony diameter of the treatment.

The lethal concentration was defined as the lowest concentration resulting in complete growth inhibition.

### 2.8 Evaluation of ZEI/GA–CIN PT for Extending the Shelf Life of Bread

Bread samples were prepared to evaluate the ability of ZEI/GA–CIN PT to extend shelf life, using the cinnamaldehyde control dispersion as the formulation comparator. The concentration applied was defined on the basis of in vitro dose–response results, corresponding to the minimum level required for complete inhibition of all tested fungi, allowing the evaluation of antifungal performance under realistic food system conditions.

The formulation, based on Sun et al. (2020), comprised 264 g wheat flour (Orquídea, Caxias do Sul, Brazil), 40 g sugar (Caravelas, Ariranha, Brazil), 14 g soybean oil (Soya, Luziânia, Brazil), 5 g active dry yeast (Fleischmann, Petrópolis, Brazil), 1.3 g salt (Zizo, Imbituba, Brazil), and 174 g water. This formulation was used because it provides a suitable substrate for fungal growth due to its post‐baking water activity (∼0.95) and nutritional composition (carbohydrates, proteins, lipids, and minerals), making it suitable for shelf‐life assessment. For antifungal treatments, 60 g of either the cinnamaldehyde control dispersion or ZEI/GA–CIN PT was incorporated into the dough. Both treatments contained the same cinnamaldehyde concentration (3.16 mg mL^−1^), corresponding to a nominal concentration of 380 mg kg^−1^ in the dough. The amount of water (114 g) was adjusted to maintain total liquid content similar to the untreated formulation.

Ingredients were mixed for 6 min in a planetary mixer (Philco, Joinville, Brazil), shaped in 24 × 10 × 5 cm^3^ pans, fermented at 30°C for 60 min, and baked in an oven (Tedesco ITT150E, Caxias do Sul, Brazil) at 200°C for 20 min. After cooling to 25°C, breads were sliced (11 × 9 × 1.5 cm^3^) and stored in polyethylene zip‐lock bags at 25°C.

Fungal growth was monitored daily by recording the number of slices showing visible contamination (*n* = 15 per group). The incidence of fungal growth was calculated as follows:

(4) $$\mathrm{Fungal}\;\mathrm{incidence}\;\left(\%\right)=\frac{Nc}{Nt}\times 100$$

where *Nc* is the number of contaminated slices and *Nt* is the total number of slices evaluated.

The extent of fungal growth was quantified by digital image analysis using ImageJ (National Institutes of Health, USA), expressed as the percentage of contaminated area, as described by Nouska et al. (2023). Images were acquired under standardized conditions using a smartphone camera (Sony, LYTIA 600, 50 MP, China) positioned perpendicular to the sample at a fixed distance (20 cm), with uniform diffuse lighting and a matte white background to minimize shadows and reflections. Image processing was performed by converting images to 8‐bit gray scale, followed by automatic thresholding (Otsu method) to segment fungal growth. The extent of fungal growth was calculated according to the following equation:

(5) $$\mathrm{Fungal}\;\mathrm{growth}\;\mathrm{extent}\left(\%\right)=\frac{Af}{At}\times \;100$$

where *Af* is the area covered by fungal growth, and *At* is the total slice area.

### 2.9 Physicochemical and Antifungal Stability of ZEI/GA–CIN PT Dispersions During Storage

The physicochemical stability and antifungal activity of ZEI/GA–CIN PT dispersions, stored in 30 mL amber vials, were evaluated during storage at 4°C ± 2°C and 25°C ± 2°C, simulating refrigerated and ambient conditions, respectively.

#### 2.9.1 Physicochemical Stability

ZEI/GA–CIN PT and blank particles (ZEI/GA PT) were evaluated at 0, 7, 30, 60, 120, and 210 days by measuring mean particle diameter, PDI, and *ζ*‐potential, as previously described in Section 2.6.1. Physicochemical characterization was discontinued after visible sedimentation was observed, as this prevented reliable DLS and *ζ*‐potential measurements.

#### 2.9.2 Effect of Storage on Antifungal Activity of ZEI/GA–CIN PT Dispersions

The effect of storage on antifungal activity was evaluated at 0, 7, 30, 60, 90, 120, 150, 180, and 210 days for the cinnamaldehyde control dispersion and ZEI/GA–CIN PT using the mycelial growth inhibition assay described in Section 2.7.2, at the lethal concentration previously determined. This more frequent sampling schedule, compared with physicochemical characterization, was adopted to identify the onset of efficacy loss for the cinnamaldehyde control dispersion and ZEI/GA–CIN PT at each storage temperature. Antifungal activity continued to be assessed after visible sedimentation prevented reliable physicochemical measurements. Results were expressed as percentage antifungal efficacy, calculated based on the inhibition of fungal growth after 7 days of incubation at 25°C.

### 2.10 Statistical Analysis

Statistical analysis was performed using GraphPad Prism (GraphPad Software, USA). Mean particle diameter, PDI, and *ζ*‐potential were compared between ZEI/GA PT and ZEI/GA–CIN PT using Student's *t*‐test. Differences were considered significant at *p* < 0.05.

For the dose–response assay, treatment (cinnamaldehyde control dispersion and ZEI/GA–CIN PT) and concentration level were the main experimental factors. Their effects, including the treatment × concentration interaction, on mycelial growth inhibition were evaluated for each fungal species using two‐way analysis of variance (ANOVA) followed by Tukey's post hoc test (*p* < 0.05).

For the physicochemical stability study, particle type (ZEI/GA PT and ZEI/GA–CIN PT) and storage time were the main experimental factors at each storage temperature. Their effects, including the particle type × storage time interaction, on mean particle diameter, PDI, and *ζ*‐potential were evaluated separately using two‐way ANOVA followed by Tukey's test (*p* < 0.05).

## 3 Results and Discussion

### 3.1 Preliminary Evaluation of the Antifungal Potential of Cinnamaldehyde

Table 1 shows the antifungal activity of cinnamaldehyde against bread spoilage fungi under both agar diffusion and vapor‐phase conditions. No visible fungal growth was observed in treated samples compared to the respective controls, indicating strong inhibitory activity of this phenylpropanoid regardless of the mode of application. These findings demonstrate that cinnamaldehyde is effective through both direct contact and its volatile phase, highlighting its potential as a natural antifungal agent for food preservation.

**TABLE 1:** Evaluation of the antifungal potential of cinnamaldehyde (CIN) under direct (agar diffusion) and noncontact (vapor‐phase) conditions.

Assay	*Penicillium citrinum*		*Penicillium roqueforti*		*Aspergillus flavus*		*Aspergillus niger*	
	Control	CIN	Control	CIN	Control	CIN	Control	CIN
**Agar diffusion**	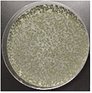	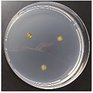	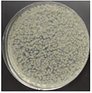	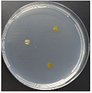	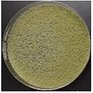	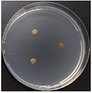	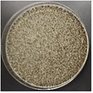	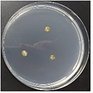
**Antifungal potential**	+		+		+		+	
	Control	CIN	Control	CIN	Control	CIN	Control	CIN
**Vapor phase**	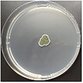	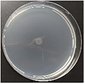	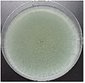	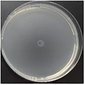	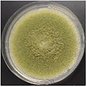	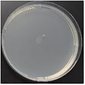	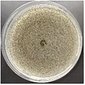	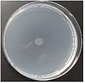
**Antifungal potential**	+		+		+		+	

*Note*: + indicates antifungal activity, defined by the absence of visible fungal growth on plates after incubation at 25°C for 7 days, relative to the corresponding controls.

The effectiveness of CIN in both assays suggests that its antifungal activity occurs via direct interaction with fungal cells as well as through volatilization, which is particularly relevant for controlling fungal contamination in bakery products (Moro et al. 2022). The antifungal activity of cinnamaldehyde under direct contact conditions has been reported against *A. flavus*, *A. niger*, and *P. citrinum* (Qu et al. 2019; Sun et al. 2020; Liu et al. 2024), whereas, for *P. roqueforti*, only cinnamon essential oil, whose major component is cinnamaldehyde, has been shown to be effective (Jeong et al. 2014).

To the best of our knowledge, this is the first study to report the antifungal activity of CIN against *P. roqueforti*, including its effectiveness in the vapor phase, highlighting its potential as a volatile antifungal agent for bakery applications. Therefore, the confirmed antifungal potential of CIN in this preliminary assessment supports the use of encapsulation strategies to protect the active compound and preserve its antifungal efficacy during bread storage.

### 3.2 Colloidal Properties and Encapsulation Performance of Zein/GA Nanoparticles

Table 2 presents the effect of cinnamaldehyde incorporation on the physicochemical properties of ZEI/GA nanoparticles. Both ZEI/GA PT (251.7 nm) and ZEI/GA–CIN PT (255.8 nm) exhibited particle sizes in the nanometric range, confirming the efficiency of the adopted nanostructuring strategy. The similar particle diameters observed before and after CIN incorporation, with no significant difference between the formulations (Student's *t*‐test, *p* = 0.838), suggest that the bioactive compound was effectively accommodated within the polymeric matrix without inducing aggregation or structural disruption. Particles within this diameter range are particularly desirable for food applications, as they offer a higher surface area, improved dispersibility in aqueous matrices, and enhanced potential to increase the bioavailability and efficacy of bioactive compounds such as CIN (Dacanal 2024).

**TABLE 2:** Physicochemical characterization of zein/gum arabic particles loaded with cinnamaldehyde (ZEI/GA–CIN PT), including mean particle diameter, polydispersity index (PDI), zeta potential (*ζ*), loading capacity (LC), and encapsulation efficiency (EE).

Particle	Mean particle diameter (nm)	PDI	*ζ* (mV)	LC (µg mg^−1^)	EE (%)
ZEI/GA PT (control)	251.7 ± 22.67A	0.14 ± 0.01B	−7.0 ± 4.6B	—	—
ZEI/GA–CIN PT	255.8 ± 23.40A	0.17 ± 0.01A	−21.8 ± 3.2A	101.4 ± 5.7	71 ± 4

*Note*: Values are expressed as mean ± standard deviation of three independent nanoparticle preparation batches (*n* = 3). Different letters within the same column indicate significant differences between ZEI/GA PT and ZEI/GA–CIN PT (Student's *t*‐test, *p* < 0.05). Empty zein/gum arabic nanoparticles (ZEI/GA PT) were used as the control.

The incorporation of cinnamaldehyde resulted in a small but statistically significant increase in the PDI, from 0.14 to 0.17 (Student's *t*‐test, *p* = 0.021). Nevertheless, both values remained below 0.2, indicating a narrow and homogeneous particle size distribution (Danaei et al. 2018). PDI values between 0.1 and 0.3 have been reported for zein/GA complexes used as wall materials, prepared by antisolvent precipitation for the encapsulation of hydrophobic compounds such as curcumin (Zhang et al. 2022; Jayalakshmi et al. 2025) and astaxanthin (Song et al. 2025).

ZEI/GA–CIN PT exhibited a higher zeta potential magnitude (*ζ* = −21.8 mV) compared to the control (*ζ* = −7.0 mV; Student's *t*‐test, *p* = 0.010), suggesting that cinnamaldehyde incorporation altered the surface charge of the particles. This change may be attributed to structural rearrangements within the polymeric matrix and increased exposure of anionic groups. Higher absolute *ζ* values are associated with stronger electrostatic repulsion between particles, contributing to improved colloidal stability (Gao et al. 2023).

Zeta potential values with magnitudes between 15 and 30 mV, whether positive or negative, are generally associated with moderately stable colloidal dispersions, further supporting the role of CIN in modulating the interfacial properties of the nanoparticles (Uskoković 2013).

The loading capacity and encapsulation efficiency values further demonstrate the suitability of the zein/GA system as a carrier for cinnamaldehyde. The loading capacity of 101.4 µg mg^−1^ indicates that a substantial amount of cinnamaldehyde was incorporated per unit mass of nanoparticles. High loading capacity is particularly relevant for food preservation applications, as it allows the delivery of effective antifungal doses with a reduced amount of carrier material (Martins et al. 2022).

The encapsulation efficiency of cinnamaldehyde reached 71%, confirming effective encapsulation within the polymeric matrix. This value is consistent with those reported for hydrophobic bioactive compounds in protein–polysaccharide nanoparticles, which typically exhibit encapsulation efficiencies above 60% (Zhang et al. 2022; Mendoza et al. 2025; Chen and Zhong 2015). The hydrophobic nature of this phenylpropanoid favors interactions with the nonpolar domains of zein, enhancing its retention within the nanoparticles, whereas electrostatic interactions and hydrogen bonding mediated by gum arabic further improve encapsulation performance (Yan et al. 2022; Jin et al. 2022).

### 3.3 Morphological, Structural, and Thermal Characterization of Zein/GA Nanoparticles

**FIGURE 2:**
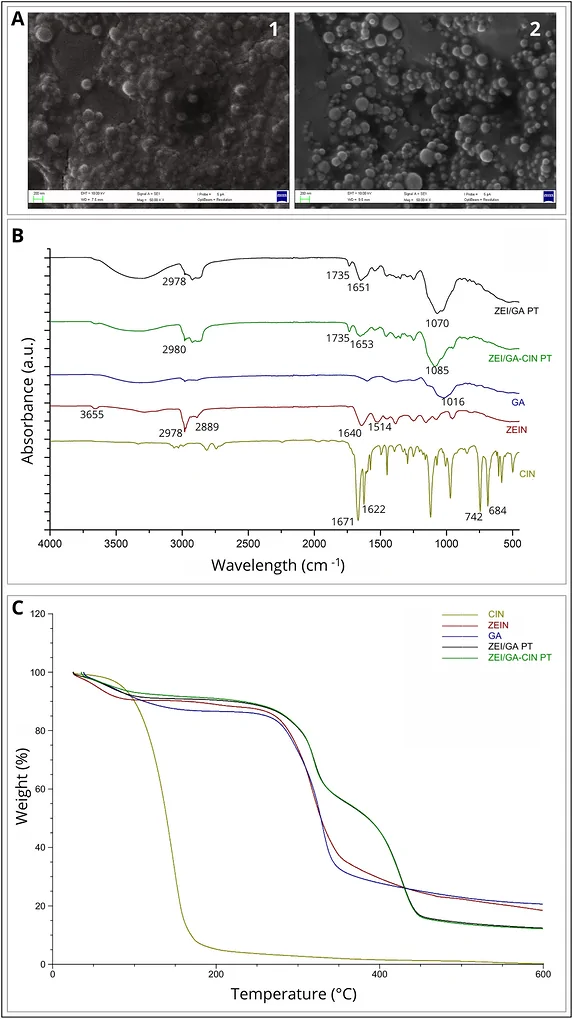
Morphological, structural, and thermal characterization of empty zein/gum arabic nanoparticles (ZEI/GA PT) and cinnamaldehyde‐loaded nanoparticles (ZEI/GA–CIN PT). (A) Scanning electron microscopy (SEM) images of ZEI/GA PT (1) and ZEI/GA–CIN PT (2). (B) Fourier‐transform infrared (FTIR) spectra and (C) thermogravimetric analysis (TGA) thermograms of CIN, ZEI, GA, ZEI/GA PT, and ZEI/GA–CIN PT. In panel C, the ZEI/GA PT and ZEI/GA–CIN PT curves are largely overlapped because of their similar thermal degradation profiles.

Figure 2 illustrates the impact of cinnamaldehyde incorporation on the morphological, structural, and thermal properties of zein/GA nanoparticles. SEM images (Figure 2A) show that both ZEI/GA PT and ZEI/GA–CIN PT exhibit similar spherical morphologies with smooth, compact surfaces, devoid of pores or cracks. This similarity indicates that cinnamaldehyde incorporation did not substantially affect particle morphology. These features are crucial for enhancing the protection and retention of CIN within the nanoparticles, given its high volatility. When encapsulated in an appropriate wall material, CIN exhibits reduced vapor‐phase permeability, thereby limiting its volatilization (da Rosa et al. 2020).

Figure 2B shows the FTIR spectra of the nanoparticle components and formulations. Cinnamaldehyde showed characteristic peaks at 1671, 1622, 742, and 684 cm^−1^, corresponding to C = O, C = C, C–H stretching vibrations, and alkenyl ring vibrations, respectively (Cui et al. 2025; Shu et al. 2025). Zein displayed peaks at 2978 cm^−1^ (N–H stretching), 1640 cm^−1^ (amide I C = O stretching), and 1514 cm^−1^ (C–N stretching), with additional peaks at 3655 and 2889 cm^−1^ attributed to –OH and C–H vibrations from amino acid residues, as previously reported by Farnad and Farhadi (2023) and Mahalakshmi et al. (2020). Gum arabic displayed a characteristic peak at 1016 cm^−1^, corresponding to C–H stretching (Mendoza et al. 2025; Al‐Idee et al. 2022). In the spectrum of ZEI/GA PT, the main bands associated with zein and gum arabic were preserved, although slight shifts in peak positions and changes in intensity were observed, indicating modifications in intermolecular interactions during nanoparticle formation. For ZEI/GA–CIN PT, the peaks of zein and gum arabic remained, whereas the cinnamaldehyde‐specific peaks were absent, confirming successful encapsulation. In addition, the gum arabic peak shifted from 1070 cm^−1^ in ZEI/GA PT to 1085 cm^−1^ in ZEI/GA–CIN PT. Zein peaks were attenuated (2978–2980 cm^−1^) and slightly shifted (1651–1735 cm^−1^), further confirming molecular interactions between cinnamaldehyde and the polymeric matrix. These spectral changes provide clear evidence of encapsulation and interactions among the nanoparticle constituents.

TGA thermograms (Figure 2C) revealed distinct degradation profiles for the individual components and the nanoparticle systems. Cinnamaldehyde exhibited rapid mass loss, with the onset of degradation at approximately 95°C and reaching about 90% mass loss by 165°C. In contrast, zein and gum arabic showed broader, multi‐step degradation patterns, indicative of their greater thermal resistance. Major mass loss for zein and gum arabic occurred between 272°C and 350°C, with reductions of approximately 68% and 64%, respectively, reflecting the thermal degradation of the protein structure of zein (Barros et al. 2024) and the polysaccharide chains of gum arabic (Cozic et al. 2009). By 600°C, both materials reached ∼80% total mass loss, consistent with the progressive breakdown of their macromolecular structures (Barros et al. 2024; Cozic et al. 2009). The thermal profiles of ZEI/GA PT and ZEI/GA–CIN PT were largely overlapping, indicating that the thermal behavior of the loaded nanoparticles was mainly governed by the zein/gum arabic matrix. In addition, ZEI/GA–CIN PT did not show the abrupt low‐temperature mass loss observed for neat cinnamaldehyde, suggesting that nanoencapsulation reduced cinnamaldehyde volatilization and/or thermal degradation under the analytical conditions used. These results support the role of the zein/gum arabic matrix in improving the thermal stability and provide effective protection for the encapsulated compound.

### 3.4 Nanostructural and Surface Characterization of Zein/Gum Arabic Nanoparticles

**FIGURE 3:**
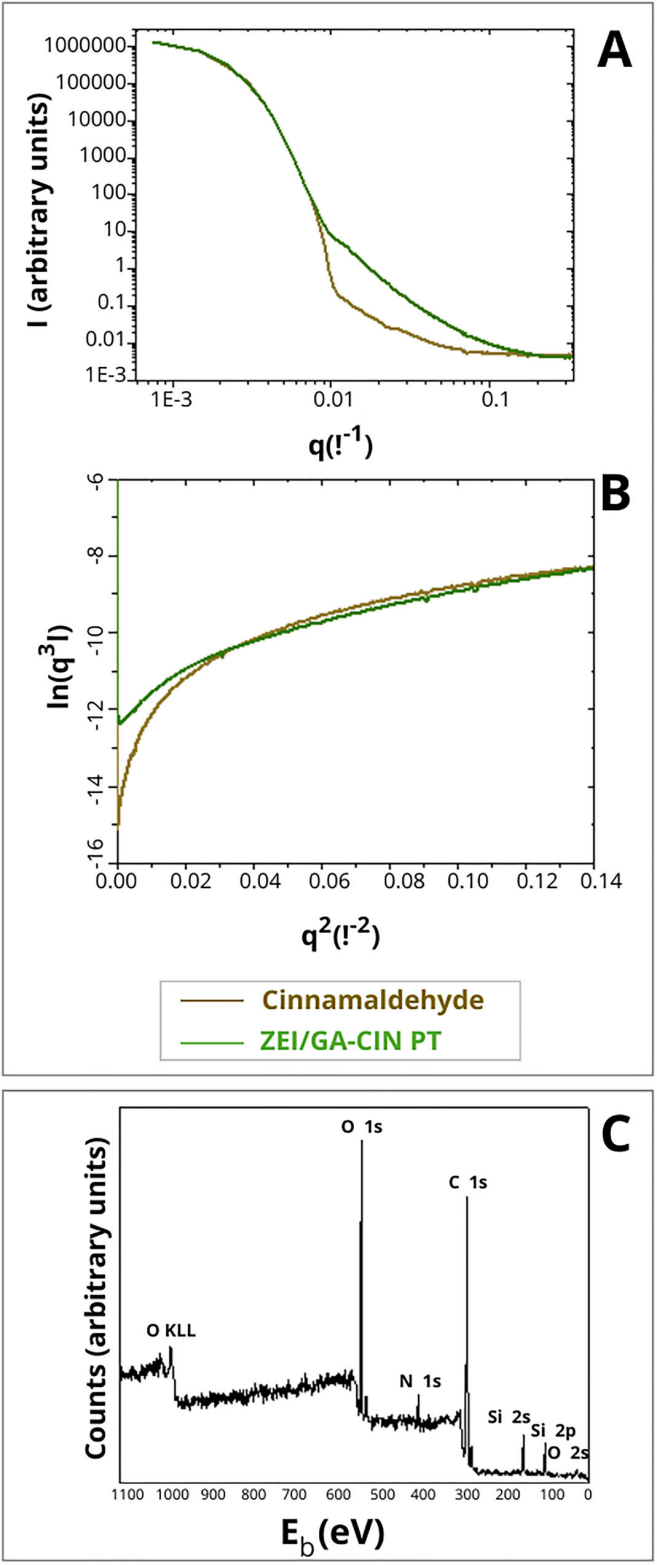
(A) Small‐angle x‐ray scattering (SAXS) patterns of cinnamaldehyde and cinnamaldehyde‐loaded zein/gum arabic nanoparticles (ZEI/GA–CIN PT); (B) the respective Porod plot of the SAXS data; and (C) x‐ray photoelectron spectroscopy (XPS) survey spectra highlighting the surface composition of ZEI/GA–CIN PT.

To further investigate the internal nanostructure and organization of cinnamaldehyde within the zein/gum arabic matrix, SAXS analysis was performed. The SAXS profiles of Figure 3 provided further insight into the nanostructural organization of cinnamaldehyde before and after encapsulation.

The scattering curve of both samples showed a rapid decay in intensity with increasing *q*, without a structure factor, suggesting a predominantly molecularly dispersed or weakly associated system (Figure 3A). This behavior indicates that cinnamaldehyde does not form periodic arrangements or highly organized domains at the nanoscale.

The scattering profiles without a defined structure factor are generally indicative of the absence of long‐range order and are consistent with amorphous or structurally disordered systems. Although SAXS does not provide direct information on specific molecular interactions, protein–polysaccharide systems, such as zein and gum Arabic, are typically stabilized by non‐covalent interactions, including hydrophobic interactions and hydrogen bonding, which contribute to their colloidal stability (Falsafi et al. 2022). Therefore, the absence of crystalline or phase‐separated domains suggests that cinnamaldehyde remained molecularly dispersed within the biopolymeric matrix. This organization may increase interfacial contact between cinnamaldehyde and the encapsulating materials, favoring intermolecular associations and reducing molecular mobility (Falsafi et al. 2022; Genix and Oberdisse 2023). This structural organization is consistent with the adequate encapsulation efficiency mentioned previously and provides a plausible nanostructural basis for the physicochemical stability of ZEI/GA–CIN PT during storage.

Differences between neat cinnamaldehyde and ZEI/GA–CIN PT became more evident in the Porod representation (Figure 3B) (Li et al. 2000). The Porod slopes were 13.6 and 15.8 for cinnamaldehyde and ZEI/GA–CIN PT, respectively. The more negative deviation observed for ZEI/GA–CIN PT is consistent with a two‐phase system with distinct electron density regions. This behavior suggests the presence of well‐defined interfaces, indicating that cinnamaldehyde is not merely dispersed in the medium but incorporated within nanoscale domains in the zein/gum arabic matrix, supporting the effectiveness of the encapsulation process. The formation of these nanoscale domains also provides a structural basis for the retention of cinnamaldehyde within the particles, suggesting that the active compound was incorporated into the zein/gum arabic matrix rather than being predominantly surface‐exposed. This organization may limit premature diffusion and volatilization while favoring a gradual release during application (Zhang et al. 2022).

Complementary XPS analysis provides more direct insight into the surface organization of the particles. The presence of nitrogen in the survey spectrum of the ZEI/GA–CIN PT, an element not found in cinnamaldehyde, confirms that this signal arises from zein. Given the surface sensitivity of XPS, this result indicates that zein is predominantly located at the particle interface, supporting the formation of an encapsulated structure. Such surface organization is consistent with an effective encapsulation process and provides a structural basis for reducing premature volatilization and diffusion of cinnamaldehyde, thereby contributing to its prolonged physicochemical stability and sustained availability during application (Zhang et al. 2022).

### 3.5 Effect of Encapsulation on the in Vitro Antifungal Activity of Cinnamaldehyde

Table 3 summarizes the impact of encapsulation within the ZEI/GA nanocarrier system on MIC and MFC of cinnamaldehyde against bread spoilage fungi. For *P. citrinum* and *A. flavus*, encapsulation did not alter antifungal performance, as identical MIC and MFC values were observed for the cinnamaldehyde control dispersion and ZEI/GA–CIN PT, indicating preservation of its bioactivity after incorporation into the zein/gum arabic matrix. For *A. niger*, encapsulation reduced the MIC from 165.0 to 82.5 µg mL^−1^, whereas the MFC remained unchanged, suggesting an enhancement of fungistatic activity without affecting fungicidal efficacy.

**TABLE 3:** Effect of encapsulation on the minimum inhibitory concentration (MIC) and minimum fungicidal concentration (MFC) of cinnamaldehyde.

Fungi	Concentration (µg mL^−1^)			
	Cinnamaldehyde^a^		ZEI/GA–CIN PT^b^	
	MIC	MFC	MIC	MFC
*Penicillium citrinum*	82.5	82.5	82.5	82.5
*Penicillium roqueforti*	82.5	82.5	41.3	41.3
*Aspergillus flavus*	165.0	165.0	165.0	165.0
*Aspergillus niger*	165.0	165.0	82.5	165.0

^a^ Cinnamaldehyde control dispersion.

^b^ Zein/gum arabic particles loaded with cinnamaldehyde.

In contrast, for *P. roqueforti*, encapsulation resulted in a reduction of both the MIC and MFC (from 82.5 to 41.3 µg mL^−1^), indicating a potentiating effect on antifungal activity. This species‐dependent response may be associated with differences among the tested fungi in cell wall architecture, plasma membrane composition, permeability, and intrinsic susceptibility to cinnamaldehyde, all of which may influence the accessibility of the encapsulated compound to its cellular targets (Gow and Lenardon 2023; Sun et al. 2020). In addition, the zein/gum arabic matrix may have contributed to this response by improving the protection and dispersion of cinnamaldehyde within the biopolymeric particles, as supported by the structural characterization and encapsulation performance observed in this study (Falsafi et al. 2022; Yan et al. 2022; Li et al. 2018; Zhang et al. 2022). Future mechanistic studies integrating release kinetics, membrane permeability assays, intracellular leakage measurements, nanoparticle–fungal cell interaction imaging, and cell wall/plasma membrane composition analyses will be necessary to discriminate among the contributions of nanoparticle adhesion, cinnamaldehyde accessibility, and intrinsic fungal susceptibility to the species‐dependent antifungal response observed here. Considering that *P. roqueforti* is the predominant fungus in bakery environments (Garcia, Bernardi, et al. 2019), encapsulation represents an effective strategy to control the main fungal spoilage agent in bread.

Overall, encapsulation maintained or enhanced the antifungal efficacy of cinnamaldehyde, depending on the fungus. Protein–polysaccharide delivery systems are widely recognized for their ability to enhance physicochemical stability, protect volatile compounds, and enable controlled release, thereby potentially improving bioavailability and facilitating interactions with microbial cells. Wang, Liu, et al. (2024) and Chen et al. (2022) demonstrated that the antifungal activity of cinnamaldehyde against *Aspergillus brasiliensis* is preserved when the gelatin and gum arabic complex is used as encapsulating material. However, the incorporation of cinnamaldehyde‐loaded zein/gum arabic nanoparticles into bread formulations has not been previously reported.

### 3.6 Effect of Encapsulation on the Dose–Response Behavior and Lethal Concentration of Cinnamaldehyde

**FIGURE 4:**
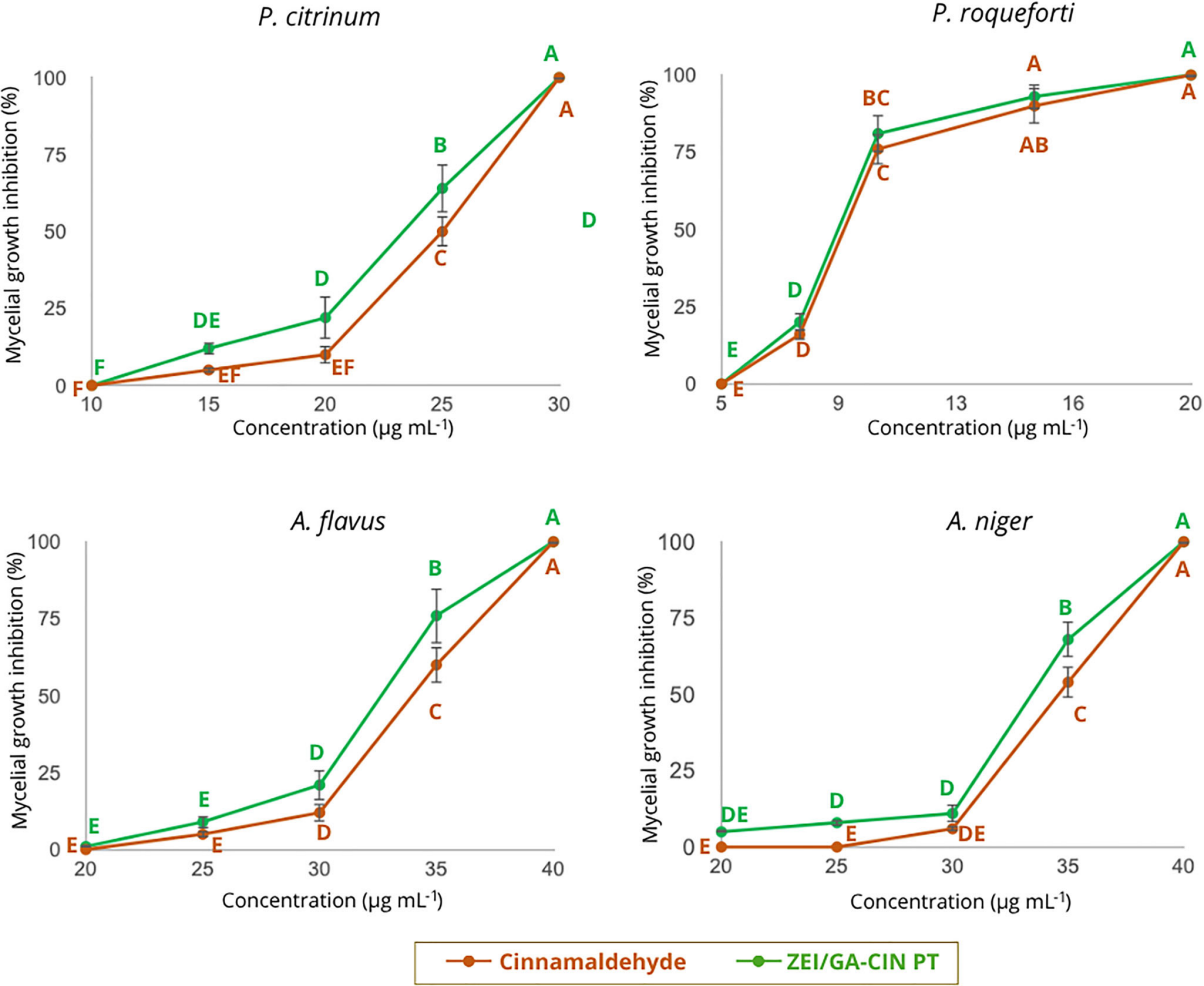
Dose–response curves of the cinnamaldehyde control dispersion (CIN) and encapsulated cinnamaldehyde (ZEI/GA–CIN PT) on mycelial growth inhibition of bread spoilage fungi. Values represent mean ± standard deviation (*n* = 9). Inhibition (%) was determined after 7 days of incubation at 25°C. Different letters indicate significant differences among treatment (CIN and ZEI/GA–CIN PT) × concentration combinations according to two‐way ANOVA followed by Tukey's multiple comparison test (*p* < 0.05). Green letters refer to ZEI/GA–CIN PT, and brown letters refer to the cinnamaldehyde control dispersion.

The dose–response curves obtained from mycelial growth inhibition assays under solid‐medium conditions provided further insight into the antifungal performance of cinnamaldehyde. As shown in Figure 4, mycelial growth inhibition increased as a function of cinnamaldehyde concentration for all fungi.

Encapsulation influenced the shape of the dose–response curves, particularly at subinhibitory concentrations. Two‐way ANOVA showed significant effects of treatment, concentration level, and their interaction (*p* < 0.05) for *P. citrinum*, *A. flavus*, and *A. niger*. ZEI/GA–CIN PT exhibited significantly higher inhibition than the cinnamaldehyde control dispersion at 20 and 25 µg mL^−1^ for *P. citrinum*, at 35 µg mL^−1^ for *A. flavus*, and at 25 and 35 µg mL^−1^ for *A. niger*. In contrast, no significant differences between the cinnamaldehyde control dispersion and ZEI/GA–CIN PT were observed for *P. roqueforti* at any concentration evaluated. A similar trend was reported by Zhang et al. (2023), who demonstrated that cinnamaldehyde microencapsulated in calcium alginate exhibited enhanced efficacy in controlling the growth of *A. flavus* in peanuts compared to its non‐encapsulated form.

Lethal concentration values varied among species, with *P. roqueforti* being the most sensitive (20 µg mL^−1^), followed by *P. citrinum* (30 µg mL^−1^), whereas *A. flavus* and *A. niger* exhibited greater tolerance (40 µg mL^−1^), highlighting interspecies differences in susceptibility. For all fungi except *P. roqueforti*, an increase of only 10 µg mL^−1^ resulted in complete inhibition of mycelial growth, corresponding to an increase of approximately 90% in antifungal activity. This pattern reflects a typical threshold effect, in which small increases above a critical concentration lead to rapid disruption of essential cellular processes and consequent growth suppression (Ji et al. 2024).

Despite the enhanced antifungal activity at subinhibitory concentrations, lethal concentration values remained unchanged across all species, indicating that encapsulation improves antifungal performance without increasing the amount required for complete growth inhibition. This behavior is particularly relevant for controlling fungal growth in food systems.

### 3.7 Evaluation of ZEI/GA–CIN PT for Extending Bread Shelf Life

**FIGURE 5:**
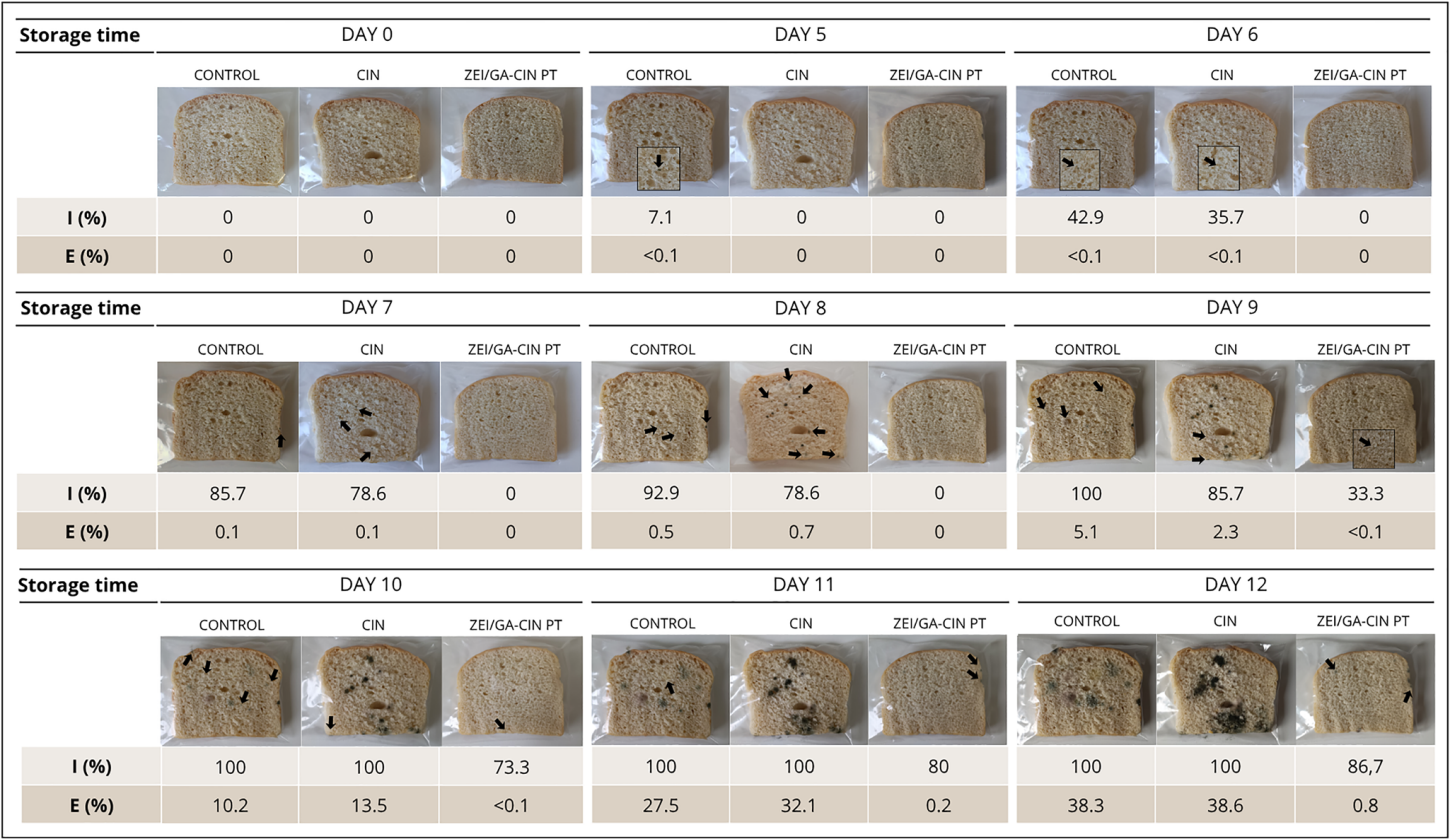
Monitoring of fungal growth on bread slices treated with the cinnamaldehyde control dispersion (CIN) or encapsulated cinnamaldehyde (ZEI/GA–CIN PT) during storage. Untreated samples consisted of bread without antifungal addition. Arrows indicate initial sites of fungal growth, and magnified images highlight colonies observed on the reverse side of the slices. Values represent fungal incidence (I) and growth extent (E), based on 15 bread slices per treatment (*n* = 15). Images are representative of each treatment group.

Following confirmation of cinnamaldehyde antifungal activity in vitro, the cinnamaldehyde control dispersion and ZEI/GA–CIN PT were evaluated in bread under more complex and realistic food system conditions. Figure 5 illustrates fungal incidence and growth extent on bread slices during storage in untreated samples and those treated with cinnamaldehyde control dispersion and ZEI/GA–CIN PT.

As storage progressed, differences between treatments became more pronounced. From Day 7 onward, untreated samples rapidly reached high contamination levels, exceeding 85% incidence, accompanied by a progressive increase in the affected area. In contrast, breads treated with cinnamaldehyde control dispersion showed a delayed but still substantial fungal development. ZEI/GA–CIN PT effectively suppressed fungal growth for a longer period, maintaining zero incidence up to Day 8 and significantly reducing both incidence and growth extent thereafter. From a practical perspective, visible fungal contamination was first detected on Day 5 in the untreated bread, on Day 6 in bread treated with cinnamaldehyde control dispersion, and only on Day 9 in bread treated with ZEI/GA–CIN PT. Therefore, encapsulation extended the mold‐free shelf life by approximately 4 days compared with the untreated control and by 3 days compared with cinnamaldehyde control dispersion treatment under the storage conditions evaluated.

It is important to note that relative humidity and bread water activity are key factors governing fungal development and may also influence antifungal performance by affecting fungal growth conditions and the availability of the active compound within the food matrix (Hernández‐Figueroa et al. 2025). In the present study, all samples were stored under identical conditions in sealed polyethylene bags, making it unlikely that humidity‐related variations accounted for the differences observed among treatments.

Even at later stages (Days 10–12), when fungal growth was observed in all treatments, breads containing ZEI/GA–CIN PT exhibited substantially lower contamination levels and minimal affected area compared to both the untreated samples and breads treated with the cinnamaldehyde control dispersion. This behavior highlights the enhanced and prolonged antifungal performance conferred by encapsulation, which may be associated with improved protection and availability of cinnamaldehyde within the food matrix (Shu et al. 2025). Because cinnamaldehyde is volatile, partial loss during baking may occur, and its exact retention in the baked bread was not quantified in the present study. Nevertheless, the TGA results (Figure 2C) support improved thermal protection after encapsulation, as ZEI/GA–CIN PT did not show the abrupt low‐temperature mass loss observed for neat cinnamaldehyde. Together with the antifungal efficacy observed in the baked bread, this indicates that the encapsulated system retained biologically effective activity after thermal processing. A detailed investigation of cinnamaldehyde release kinetics will provide further mechanistic insight into the relationship between compound release and the prolonged antifungal performance observed in bread.

Therefore, cinnamaldehyde was effective in extending bread shelf life, with encapsulation markedly improving its ability to delay fungal onset and limit growth progression under realistic storage conditions. This study demonstrates the feasibility of using cinnamaldehyde as a direct antifungal agent in bread, highlighting its potential as a naturally derived antifungal alternative for bread preservation. Until now, applications of this phenylpropanoid in bread preservation have been limited to active packaging or as an adjunct to conventional preservatives (Sun et al. 2020; Tan et al. 2025). Previous work with essential oils, such as thyme, cinnamon, clove, and bay leaf, achieved modest shelf‐life extensions of 2–5 days but was constrained by sensory and technological limitations (Hernández‐Figueroa et al. 2025; Silveira et al. 2026; Debonne et al. 2018). In contrast, encapsulation of cinnamaldehyde may overcome these limitations. Preliminary observations indicated no noticeable aroma changes in breads treated with ZEI/GA–CIN PT, whereas a faint characteristic odor was detected in breads treated with the cinnamaldehyde control dispersion. However, as these observations were not part of a formal sensory analysis, no definitive conclusions can be drawn regarding the impact of cinnamaldehyde on bread aroma, flavor, texture, or consumer acceptance. Therefore, although the present results demonstrate the potential of encapsulated cinnamaldehyde as a strategy for extending bread shelf life, further studies addressing cinnamaldehyde release kinetics, dose optimization, bread quality parameters, and structured sensory evaluation are required to fully assess its technological feasibility for bread preservation.

### 3.8 Stability of ZEI/GA–CIN PT Dispersion During Storage

Given the demonstrated effectiveness of ZEI/GA–CIN PT under realistic food conditions, evaluating its physicochemical and antifungal stability during storage is essential to further assess the practical applicability of this system. Therefore, the dispersion was monitored under two temperature conditions, 4°C (refrigeration) and 25°C (room temperature), to determine the influence of storage conditions on system integrity, colloidal stability, and antifungal performance over time.

#### 3.8.1 Physicochemical Stability

Table 4 summarizes the physicochemical stability of ZEI/GA–CIN PT and ZEI/GA PT dispersions during storage. Under refrigerated storage (4°C), both ZEI/GA PT and ZEI/GA–CIN PT dispersions maintained stable mean particle diameter throughout the 210‐day storage period. Mean particle diameter remained statistically unchanged, and PDI values were consistently below 0.2 up to 120 days, indicating the preservation of a narrow size distribution and colloidal homogeneity. At 210 days, ZEI/GA–CIN PT showed a significant reduction in PDI.

**TABLE 4:** Physicochemical stability of cinnamaldehyde‐loaded zein/gum arabic nanoparticles (ZEI/GA–CIN PT) during storage at 25°C ± 2°C and 4°C ± 2°C, compared with empty nanoparticles (ZEI/GA PT).

Temperature	Particle	0 days	7 days	30 days	60 days	120 days	210 days
**Mean particle diameter (nm)**							
4°C	ZEI/GA PT	251.7 ± 22.67Aa	258.1 ± 18.78Aa	232.7 ± 23.73Aa	245.4 ± 20.41Aa	247.2 ± 23.42Aa	259.2 ± 22.9Aa
	ZEI/GA–CIN PT	255.8 ± 23.40Aa	255.6 ± 21.42Aa	266.3 ± 25.82Aa	260.6 ± 24.56Aa	259.8 ± 22.49Aa	261.3 ± 20.4Aa
25°C	ZEI/GA PT	251.7 ± 22.67Aa	263.6 ± 25.56Aa	402.9 ± 33.94Ab	468.7 ± 43.30 Abc	583.4 ± 50.76Ac	NP
	ZEI/GA–CIN PT	255.8 ± 23.40Aa	274.8 ± 22.57Aa	513.3 ± 34.5Ab	524.9 ± 48.6Ab	815.4 ± 66.97Bc	NP
**PDI**							
4°C	ZEI/GA PT	0.14 ± 0.01Aa	0.15 ± 0.00Aa	0.14 ± 0.01Aa	0.15 ± 0.00Aa	0.13 ± 0.01Aa	0.14 ± 0.01Aa
	ZEI/GA–CIN PT	0.17 ± 0.01Bab	0.16 ± 0.01Aac	0.17 ± 0.01Bab	0.19 ± 0.01Bb	0.14 ± 0.00Ac	0.08 ± 0.00Bd
25°C	ZEI/GA PT	0.14 ± 0.01Aa	0.10 ± 0.00Aa	0.13 ± 0.01Aa	0.14 ± 0.01Aa	0.27 ± 0.05Ab	NP
	ZEI/GA–CIN PT	0.17 ± 0.01Aab	0.12 ± 0.00Aa	0.12 ± 0.00Aa	0.15 ± 0.00Aab	0.18 ± 0.02Bb	NP
***ζ*‐potential (mV)**							
4°C	ZEI/GA PT	−7.0 ± 0.7Aa	−3.5 ± 0.2Aa	−18.2 ± 1.1Ab	−16.2 ± 1.5Ab	−15.8 ± 1.3Ab	−28.0 ± 2.4Ac
	ZEI/GA–CIN PT	−21.8 ± 2.5 Ba	−23.3 ± 2.4Ba	−25.5 ± 2.5Ba	−11.3 ± 1.6Ab	−9.5 ± 0.9Bb	−9.2 ± 0.9Bb
25°C	ZEI/GA PT	−7.0 ± 0.7Aa	−14.1 ± 1.0Ab	−6.1 ± 0.5Aa	−13.4 ± 1.1Ab	−8.6 ± 0.9Aa	NP
	ZEI/GA–CIN PT	−21.8 ± 2.5Ba	−8.5 ± 0.7Bb	−6.4 ± 0.6Ab	−8.2 ± 0.8Bb	−8.9 ± 0.7Ab	NP

*Note*: Values are expressed as mean ± standard deviation of three independent nanoparticle preparations (*n* = 3). Different lowercase letters indicate significant differences among storage times within the same particle type, whereas different uppercase letters indicate significant differences between particle types at the same storage time. Comparisons were performed separately at each storage temperature using two‐way ANOVA followed by Tukey's multiple comparison test (*p* < 0.05). Measurements at 210 days for samples stored at 25°C were not performed due to sedimentation of the dispersions.

Abbreviations: NP, not performed; PDI, polydispersity index.

A significant reduction in |*ζ*| was observed only after 60 days of storage at 4°C for ZEI/GA–CIN PT dispersions, whereas blank particles exhibited an initial fluctuation at Day 7, followed by more negative *ζ*‐potential values from Day 30 onward, with the greatest magnitude observed at 210 days. Although the *ζ*‐potential of ZEI/GA–CIN PT decreased significantly after 60 days of refrigerated storage, mean particle diameter remained unchanged and PDI remained below 0.2 throughout storage, indicating that this variation was not accompanied by measurable colloidal destabilization under the evaluated conditions. In protein–polysaccharide systems such as zein/gum arabic nanoparticles, colloidal stability may result from combined electrostatic and steric stabilization mechanisms rather than from electrostatic repulsion alone. This behavior is likely associated with surface rearrangements, ion adsorption, and changes in the ionization state of functional groups, which can alter the apparent surface charge without compromising overall colloidal stability (Németh et al. 2022; Shehzad et al. 2024). Notably, opposite trends were observed for the two formulations. For ZEI/GA PT, the progressively more negative *ζ*‐potential may reflect increased exposure of negatively charged gum arabic groups at the particle surface. In contrast, the reduction in |*ζ*| observed for ZEI/GA–CIN PT after 60 days may result from molecular rearrangements within the nanoparticle matrix, leading to partial shielding of surface‐charged groups. As mean particle diameter remained unchanged and PDI values were below 0.2 throughout refrigerated storage, these changes likely reflect modifications at the particle interface rather than colloidal destabilization.

In contrast, storage at 25°C markedly compromised colloidal stability. A pronounced increase in particle diameter was observed after 30 days for both systems, corresponding to an increase of over 60% in mean particle diameter and reaching values above 800 nm for ZEI/GA–CIN PT at 120 days. This behavior reflects progressive aggregation, consistent with the reduction in *ζ*‐potential magnitude over time. Indeed, sedimentation was observed at advanced storage times under these conditions, preventing further measurements at 210 days. At this temperature, *ζ*‐potential values remained low in magnitude and fluctuated throughout storage for both systems, indicating limited electrostatic stabilization. ZEI/GA–CIN PT exhibited a rapid reduction in |*ζ*| after 7 days, followed by minor variations, whereas ZEI/GA PT showed oscillatory behavior without a clear stabilization trend. Under these conditions, reduced electrostatic repulsion favors particle–particle interactions, leading to aggregation, flocculation, and eventual sedimentation. This behavior reflects the key role of interfacial interactions and surface charge in governing the colloidal stability of zein/gum arabic nanoparticles (Li et al. 2018). Changes in environmental conditions can induce polymer chain rearrangements and reduce *ζ*‐potential, thereby weakening electrostatic repulsion and promoting particle aggregation. Song et al. (2025) observed increased particle size and changes in PDI in astaxanthin‐loaded zein/gum arabic nanoparticles during storage at room temperature, attributing these effects to flocculation and partial sedimentation.

Temperature played a decisive role in nanoparticle stability. Refrigeration preserved nanometric size, surface charge, and dispersion quality, whereas ambient storage accelerated destabilization. Under refrigerated conditions, ZEI/GA–CIN PT remained structurally stable, supporting its application as an antifungal delivery system in food matrices.

#### 3.8.2 Effect of Storage on Antifungal Activity of ZEI/GA–CIN PT Dispersion

**FIGURE 6:**
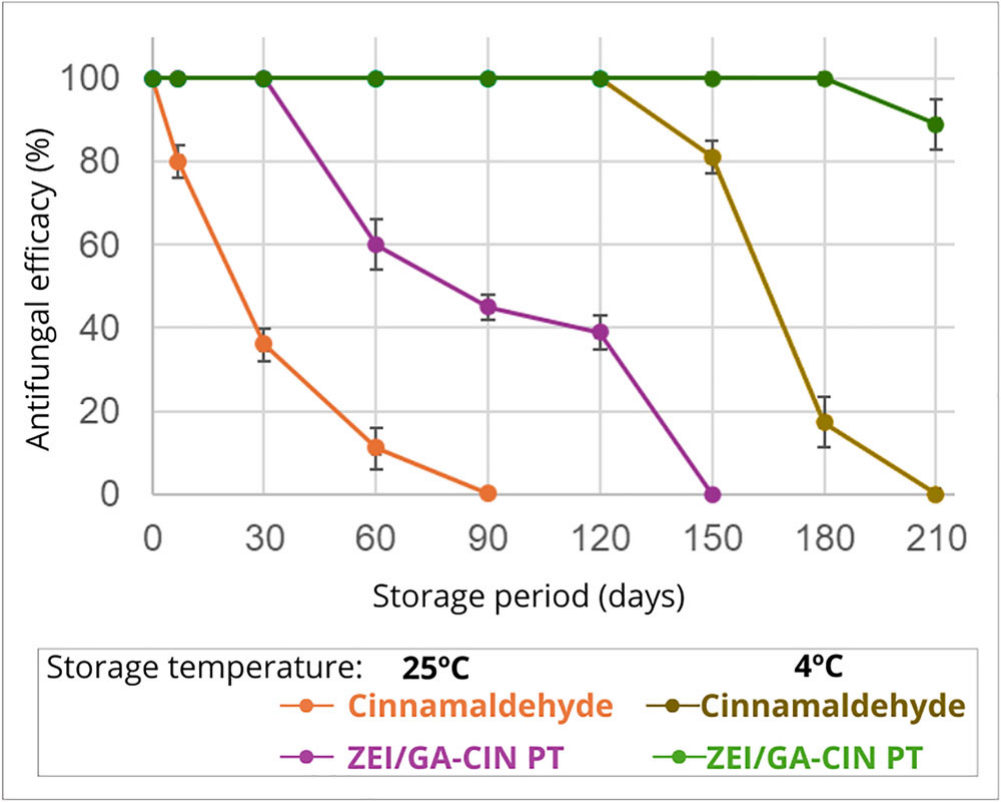
Effect of storage temperature on the antifungal activity of the cinnamaldehyde control dispersion and ZEI/GA–CIN PT against *Aspergillus niger*. Values represent mean ± standard deviation (*n* = 9).

Figure 6 shows the antifungal activity of the cinnamaldehyde control dispersion and ZEI/GA–CIN PT during storage at 4°C and 25°C. This evaluation was designed to assess the ability of encapsulation to preserve antifungal efficacy over time. The assays were conducted using *A. niger*, selected based on its higher tolerance to cinnamaldehyde, as evidenced by the lethal concentration values determined in Section 3.6, and its frequent occurrence in bread, raw materials, and processing environments (Garcia, Bernardi, et al. 2019; Garcia, Sonnenstrahl Bregão, et al. 2019). The use of this species provides a stringent and application‐relevant model for evaluating the persistence of antifungal activity under storage conditions.

At 25°C, the cinnamaldehyde control dispersion exhibited a rapid loss of efficacy, with an approximately 20% reduction in antifungal performance within the first 7 days and complete inactivation after 90 days. In contrast, ZEI/GA–CIN PT showed a more gradual decline, with an approximate 40% reduction between 30 and 60 days, reaching about 60% loss at 120 days and complete inactivation only after 150 days. This delayed loss of activity indicates that encapsulation effectively mitigates the rapid deactivation of cinnamaldehyde under ambient conditions, although it does not fully prevent long‐term deterioration.

Storage at 4°C markedly improved the preservation of antifungal activity, underscoring temperature as a key factor governing functional stability. Under these conditions, the cinnamaldehyde control dispersion remained active for a longer period but still showed an approximately 20% reduction between 120 and 150 days, followed by complete loss of activity at 210 days. In contrast, ZEI/GA–CIN PT maintained near‐complete antifungal efficacy throughout storage, with only a slight reduction (∼10%) observed between 180 and 210 days. This sustained performance demonstrates that encapsulation, combined with refrigeration, effectively preserves cinnamaldehyde bioactivity over extended storage periods, reinforcing its potential as a robust antifungal delivery system for food applications.

Despite the excellent physicochemical stability of ZEI/GA–CIN PT dispersions under refrigeration throughout the 210 days (Section 3.8.1), a slight decline in antifungal performance was observed only at prolonged storage times (180–210 days). This decoupling between structural integrity and bioactivity indicates that long‐term performance is governed not only by colloidal stability but also by the availability of the active compound. The slight reduction in efficacy is consistent with sustained‐release behavior reported for encapsulated bioactives, in which gradual depletion of the bioavailable fraction occurs over time, rather than with structural destabilization of the carrier system (Rezagholizade‐Shirvan et al. 2024).

When considering the practical applicability of ZEI/GA–CIN PT, it is important to recognize that polysorbate 80 was used as an emulsifier in the nanoparticle formulation. Although food‐grade, this synthetic emulsifier represents a limitation under a particularly strict interpretation of *clean label*, which favors formulations composed exclusively of naturally derived ingredients. Polysorbate 80 enabled the production of stable nanoparticles under the conditions evaluated. However, future studies may investigate naturally derived alternatives, such as soy lecithin or quillaja saponin (Riquelme et al. 2019; Zhu et al. 2019), to determine whether nanoparticles with comparable colloidal stability, encapsulation efficiency, and antifungal performance can be obtained.

## 4 Conclusion

This study demonstrates the feasibility and effectiveness of cinnamaldehyde‐loaded zein/gum arabic nanoparticles as an antifungal system for bread preservation. The zein/gum arabic matrix proved to be an efficient delivery platform for cinnamaldehyde, representing a robust and scalable alternative to conventional synthetic preservatives.

The developed system exhibited suitable physicochemical characteristics, including nanometric particle size, narrow size distribution, suitable colloidal stability, and encapsulation efficiency, confirming its capacity to effectively incorporate and retain cinnamaldehyde within the polymeric matrix.

Encapsulation preserved or enhanced the antifungal activity of cinnamaldehyde against *P. citrinum*, *P. roqueforti*, *A. niger*, and *A. flavus*, particularly at subinhibitory concentrations, while maintaining the lethal concentrations required for complete fungal inhibition. In situ application in bread confirmed its practical relevance, significantly delaying fungal onset and reducing contamination levels compared to both untreated bread and the cinnamaldehyde control dispersion treatment.

Storage studies further highlighted the robustness of the system. Under refrigeration, ZEI/GA–CIN PT dispersions maintained physicochemical stability and fully preserved antifungal efficacy for up to 180 days. In contrast, the cinnamaldehyde control dispersion exhibited a markedly faster loss of antifungal activity, both at ambient temperature and under refrigeration, underscoring the combined role of encapsulation and temperature in preserving functional performance.

Therefore, these findings support zein/gum arabic nanoparticles as an effective and stable carrier system for cinnamaldehyde, with potential application as a delivery system for a naturally derived antifungal compound in bread production. Further studies should address structured sensory evaluation, bread quality parameters, and cinnamaldehyde retention after baking.

## Author Contributions

**Mônia Sartor**: conceptualization, methodology, investigation, formal analysis, Writing – original draft. **Vanessa Teixeira da Rosa**: investigation, methodology, formal analysis. **Laura Smaniotto Nascimento**: investigation, formal analysis. **Alisson Steffli Thill**: investigation, formal analysis. **Fabiano Bernardi**: investigation, formal analysis. **Flávio Fonseca Veras**: methodology, conceptualization, supervision, formal analysis, writing – review and editing. **Juliane Elisa Welke**: conceptualization, writing – original draft, writing – review and editing, investigation, project administration, funding acquisition, validation, supervision, resources.

## Funding

This work was supported by the Coordination for the Improvement of Higher Education Personnel (CAPES), the Foundation for Research Support of the State of Rio Grande do Sul (FAPERGS), and the National Council for Scientific and Technological Development (CNPq, Grant 304886/2022‐0).

## Conflicts of Interest

The authors declare no conflicts of interest.

## Data Availability

The data that support the findings of this study are available from the corresponding author upon request.
